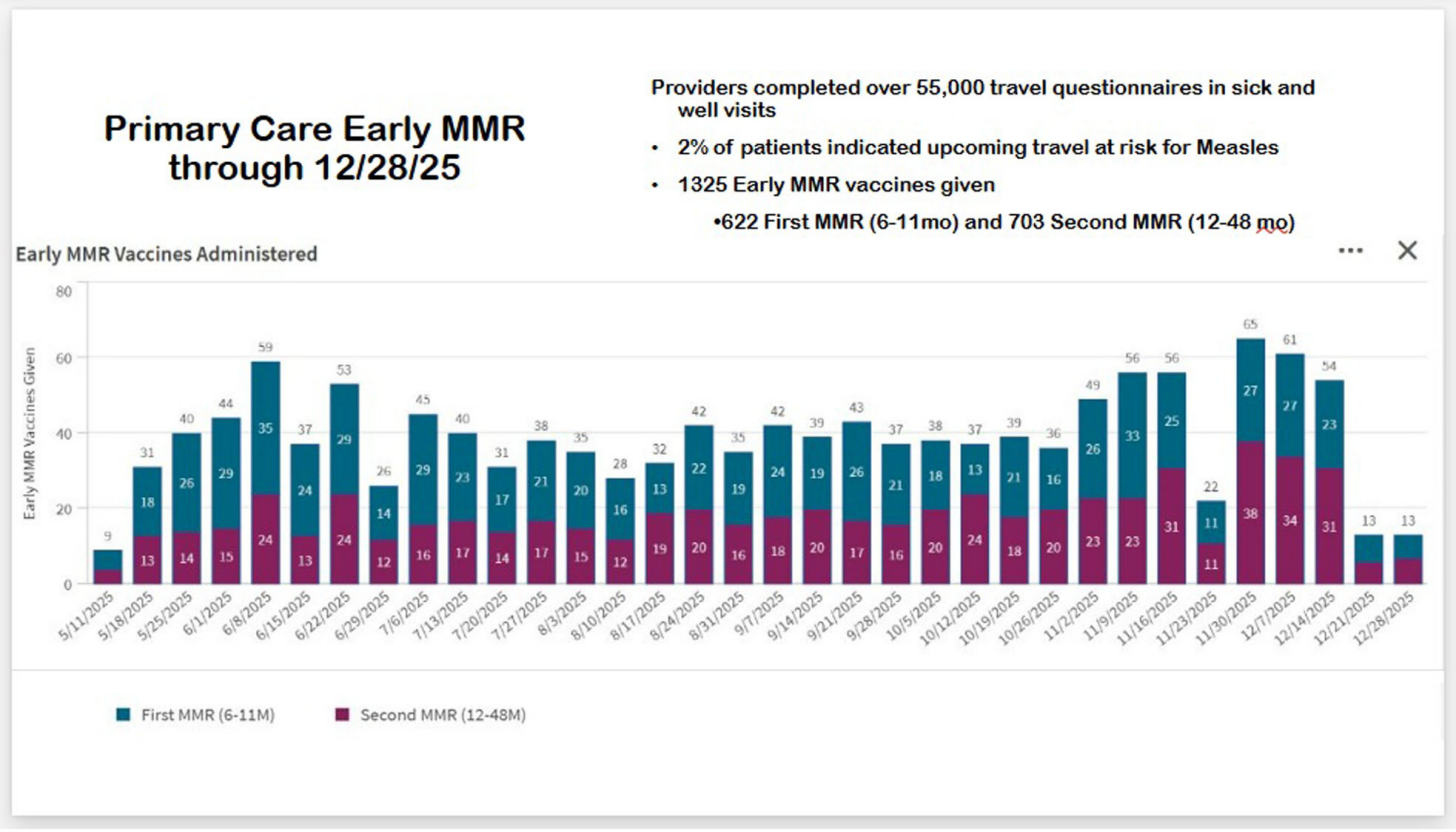# 38 Impact of Quality Assessment of Cleaning on Hospital Associated Infection Rates Using Digital Tool in Acute Care Hospital in Slovakia

**DOI:** 10.1017/ash.2026.10479

**Published:** 2026-06-23

**Authors:** Ericka Hayes, Keri Cohn, Logan Grimes, Hee-Won Yoon, Jenna Ercolani, Susan Ditaranto, Tomaine Scott, Kathleen Filograna, Lisa Biggs, Daneen Smith, Deborah Smith, Regina Toto, Matthew Butler, Julia Sammons, Sarah Smathers

**Affiliations:** 1 Univ Pennsylvania/Children’s Hospital of Philadelphia; 2 The Children’s Hospital of Philadelphia; 3 Children’s Hospital of Philadelphia; 4 CHOP; 5 The Childrens Hospital of Philadelphia

## Abstract

**Background:** The U.S. is on track to lose measles elimination status after experiencing the highest number of cases in <30 years. With ongoing transmission and falling vaccination rates, health systems must prepare for measles, focusing on minimizing secondary cases and maximizing prevention. We describe a taskforce approach for comprehensive measles (with a lens towards all-hazard)preparedness across our enterprise. **Methods:** Our mitigation and preparedness taskforce included four workstreams (Figure 1): Early MMR vaccination for children with international travel: Consistently offer and administer initial or second MMR vaccine, as appropriate, for infants and children travelling abroad. Optimize exposure and testing management: Develop tools to expedite the identification of exposed non-immune patients and optimize processes for high volume testing and result tracking. Optimize screening processes at points of entry (POE): Develop streamlined processes for reliable travel and exposure screening at all enterprise wide POEs. Contingency planning for larger scale exposure events/community spread: Develop post-exposure prophylaxis (PEP) delivery procedures to respond to large-scale community exposure events and establish partnerships with local public health (PH) departments. **Results:** Increase in vaccination for non-immune travelers in primary care: We built an EMR (electronic medical record) prompt into all child visits to screen for travel, which interfaced with vaccine clinical decision support to offer/administer vaccines. From May through 12/28/25 we administered 1325 early MMRs to travelling children (Figure 2). EMR-based report to identify exposed individuals and their immune status: This report allows all sites to rapidly identify patients needing PEP after exposure. We also developed processes for pre-emptive approval for large scale testing, result tracking, and high-volume patient notification. Optimization of Screening and POE with EMR upgrades, PPE kits: We are upgrading our EMR to better integrate travel & exposure screening. We developed additional resources including screening supports for non-English languages and PPE kits for immediate use at POE when patients screen positive. Development of PEP clinics and PH Collaboration: We developed processes to stand up PEP clinics at our hospitals to expedite large volume PEP administration. We worked with PH to outline roles, capabilities and resources for response to community outbreaks, optimized staff fit testing and developed drills across all care settings. **Conclusions:** Preparedness for highly contagious infectious diseases and emerging PH threats is critical. A taskforce approach provides a structured framework for collaboration and mobilization of resources. By utilizing an all-hazards lens, this framework is broadly applicable for vaccine preventable infections and other infectious threats.

**Figure:**
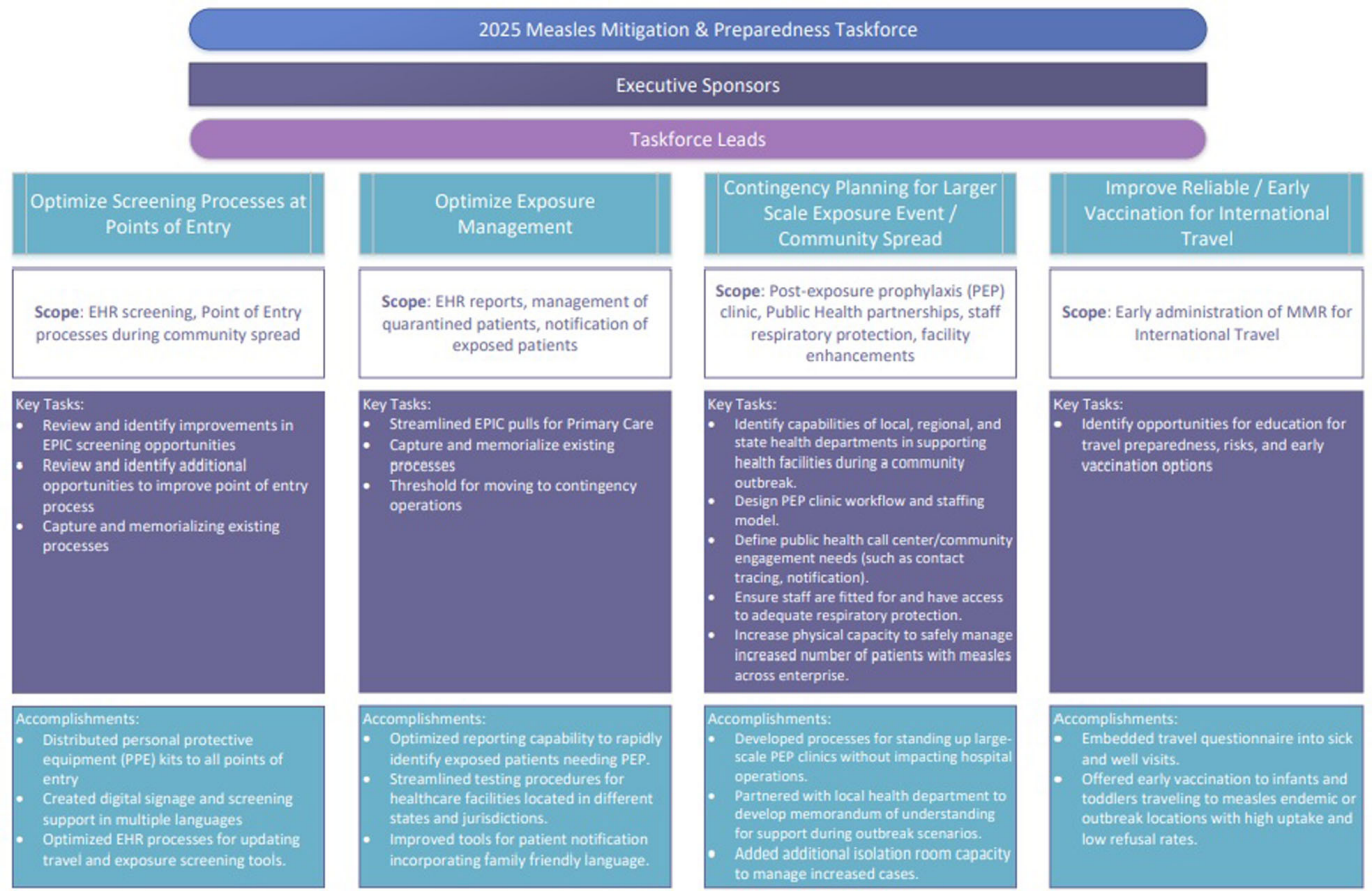

**Figure:**